# Apathy, Novelty Processing, and the P3 Potential in Parkinson’s Disease

**DOI:** 10.3389/fneur.2016.00095

**Published:** 2016-06-23

**Authors:** David A. S. Kaufman, Dawn Bowers, Michael S. Okun, Ryan Van Patten, William M. Perlstein

**Affiliations:** ^1^Department of Psychology, Saint Louis University, St. Louis, MO, USA; ^2^Department of Neurology, University of Florida, Gainesville, FL, USA; ^3^UF Center for Movement Disorders and Neurorestoration, University of Florida, Gainesville, FL, USA; ^4^Department of Clinical and Health Psychology, University of Florida, Gainesville, FL, USA; ^5^Department of Psychiatry, University of Florida, Gainesville, FL, USA; ^6^VA RR&D Brain Rehabilitation Research Center of Excellence, Malcom Randall Veterans Administration Medical Center, Gainesville, FL, USA

**Keywords:** Parkinson’s disease, apathy, novelty, ERP, P3

## Abstract

Parkinson’s disease (PD) is characterized by deficits in goal-directed behavior as well as mood and motivational symptoms, including apathy, depression, and anxiety. The present study investigated novelty processing in PD, using event-related potentials (ERPs) to characterize electrophysiological reflections of visual novelty processing. Since apathy has been associated with decreased novelty processing (P3 potentials) in highly apathetic PD patients, we were particularly interested to see if this relationship exists in a sample of PD patients with heterogeneous levels of apathy. Non-demented patients with PD receiving dopaminergic treatment (*n* = 14) and healthy control participants (*n* = 12) completed a three-stimulus oddball task while EEG was recorded. Relative to controls, the PD patients exhibited reductions in centrofrontally distributed P3 potentials when viewing novel distracters during this task. Distracter-related P3 amplitudes evoked by novel distracters were strongly associated with apathy symptoms, even after controlling for the effects of depression, anxiety, and executive function. Executive dysfunction was also predictive of novelty-related P3 processing, yet this relationship was independent from that of apathy. These findings suggest that the brain’s electrophysiological response to novelty is closely related to both motivational and cognitive symptoms in PD, even for patients whose apathy symptoms are not excessive. These results have significant implications for our understanding of non-motor symptoms in this clinical population.

## Introduction

Along with its hallmark motor symptoms, Parkinson’s disease (PD) has long been recognized for its associated cognitive and emotional deficits. Approximately 25–30% of PD patients develop dementia (1), yet even PD patients without dementia typically experience cognitive deficiencies. It has long been shown that non-demented PD patients struggle to process novel input that requires flexibility and planning (2), initiate goal-directed behavior (3), and monitor current behavior (4). Recent electrophysiological studies have found that certain measures of spontaneous EEG may serve as important biomarkers for cognitive decline in PD (5–7).

In addition to motor and cognitive dysfunction, more than 50% display symptoms of apathy (8, 9) and ~40–50% of PD patients manifest symptoms of depression (10–12). Mood dysregulation in PD appears to consist of dissociable factors of apathy, dysphoria, anhedonia, and somatic complaints (13). Mood symptoms are among the key factors leading to decreased quality of life in this population (14, 15), yet there is little neurophysiological evidence to link these symptoms to cognitive deficits in PD.

### Apathy in PD

Apathy is viewed as a lack of motivation that includes cognitive, affective, and behavioral components (16). In PD, apathy can manifest as decreased initiative to act or carry out activities (behavioral apathy), decreased reactivity and physiological blunting (affective apathy), and decreased interest in goals and planning (cognitive apathy). Early reports found that 12% of PD patients presented with apathy without depression as their primary symptom (9), while this number has been estimated to be as high as 29% (17). Apathy has been associated with executive dysfunction in PD (8, 18). Highly apathetic PD patients tend to exhibit poorer executive functions and heightened levels of depression and anxiety compared to patients with low levels of apathy (19); however, these findings appear to be influenced by the presence of dopaminergic treatment. While some patients appear to improve their apathy symptoms by taking dopaminergic medications, those with DOPA-resistant apathy demonstrate structural changes in the basal ganglia, including atrophy of the nucleus accumbens and head of the caudate (20).

### Novelty Processing in PD

Efficient processing of novelty is critical to goal-directed behavior because it drives the flexible allocation of attention that facilitates adaptation to changing environmental demands. Novel events can serve as either potential sources of engagement or as irrelevant distracters that should be ignored. Event-related potential (ERP) methods can be used to track the process of attentional orienting, which measures the cognitive resources directed toward deviant events that are unique or unexpected. When using ERPs to examine novelty processing, distracter-related P3 potentials (also known as P3a or novelty P3) have frequently been observed with a frontal or central scalp distribution (21, 22). In these paradigms, P3 potentials are evoked most prominently by infrequent, task-irrelevant distracters. Accordingly, this distracter-related P3 component has often been interpreted as reflecting an automatic, orienting reflex toward unexpected, novel events (23), which is believed to be a fundamental biological mechanism that influences exploratory behavior (24).

With regard to novelty processing, Tsuchiya et al. (25) found that PD patients exhibited reduced P3 responses to novel distracters, and these P3 reductions correlated with decreased performance on a modified version of the Wisconsin Card Sorting Test (WCST). This finding suggests that novelty processing deficits in PD may be related to executive functioning deficits that are often seen in this population (26). More recently, frontocentral P3 amplitude has been associated with disease duration and severity in PD (27). Additionally, PD patients with clinically elevated levels of apathy have demonstrated reduced electrophysiological reflections of novelty processing over frontal electrodes, relative to patients with low levels of apathy (19). Indeed, it appears that many neurological populations demonstrate a relationship between apathy and novelty ERPs, including Alzheimer’s disease (28), cortical stroke (29, 30), and subcortical stroke (31).

### Overview of the Current Study

The purpose of the present study was to examine ERP correlates of novelty processing in PD and healthy controls in order to further examine how apathy symptoms are associated with disrupted allocation of attention in PD patients. To do so, we used a visual three-stimulus oddball task and manipulated the distracter type. This approach enabled us to investigate effects of distracter novelty on ERP reflections of visual novelty. Unlike other investigators (19), we wanted to test the hypothesis that apathy is related to reduced novelty processing in a heterogeneous sample of PD patients, including those with and without elevations in apathy. We predicted that increased apathy symptoms in medicated PD patients would be associated with reduced reflections novelty processing, regardless of the specific level of apathy reported. In line with previous research, associations between novelty processing and executive dysfunction were also expected.

## Materials and Methods

### Participants

Fourteen non-demented PD patients and 12 age-matched controls participated in the current study. All patients had a diagnosis of idiopathic PD by a movement disorders specialist according to the UK Brain Bank criteria. Exclusion criteria included a history of severe psychiatric illness (i.e., schizophrenia, current depression episode, etc.), neurologic disorders affecting the brain other than PD (e.g., traumatic brain injury, stroke, tumor), severe sensory deficits, and scores below 26 on the mini-mental state examination [MMSE; (32)]. Cognitive, emotional, and electrophysiological testing sessions were performed while patients were on dopaminergic medications.

Table 1 depicts a summary of demographic, disease severity, neurocognitive, and mood information. Overall, participants were predominantly male and ranged in age from 35 to 77 years. The control group had a trend toward higher levels of education (*p* > 0.08), but did not differ on any other demographic variable. Patients performed more poorly on measures of executive function and endorsed more symptoms of depression and anxiety than controls. Although the two groups were statistically matched on apathy scores, PD patients had a greater range (0–25) than controls (3–18). The majority of individuals scored below the clinical cut-off score for apathy (<14) in both the PD (79%) and control (92%) groups. In terms of disease characteristics, the PD patients were in the early-to-mid range of disease severity, based on Hoehn–Yahr (H–Y) staging (33) and motor scores from the United Parkinson Disease Rating Scale [UPDRS; (34)] obtained within 12 months of testing. Sixty-two percent of PD patients were in H–Y stage 2, 15% were in stage 2.5, 15% were in stage 3, and 7% were in stage 4. Disease duration ranged from 5 to 15 years.

**Table 1:** **Demographic and neuropsychological data for healthy controls and PD patients**.

	Controls (*n* = 12)		PD patients (*n* = 14)		
	Mean	SD	Mean	SD	*p*
**Demographics**					
Age (years)	61.7	11.8	63.3	9.1	ns
Education (years)	16.0	3.0	13.7	3.4	ns
Female (%)	42	–	21	–	ns
Right-handed (%)	92	–	100	–	ns
**Emotional functioning**					
AS	7.7	4.1	9.7	8.3	ns
BDI-II	2.7	3.3	11.7	8.2	<0.01
STAI – trait	28.1	6.5	37.5	12.0	<0.05
STAI – state	28.5	6.5	37.9	10.8	<0.05
**Cognitive functioning**					
MMSE	28.7	1.3	29.2	1.1	ns
Dementia Rating Scale	–	–	136.4	6.9	
Boston Naming Test	56.7	4.1	56.7	2.5	ns
COWA (FAS)	40.0	10.4	37.9	15.8	ns
Semantic fluency (animals)	21.9	4.6	19.3	7.0	ns
Digit span forward (WAIS-III)	7.1	1.3	7.1	1.0	ns
Digit span backward (WAIS-III)	5.3	1.5	5.7	1.5	ns
Trails A (s)	27.7	9.4	47.2	26.3	<0.05
Trails B (s)	68.0	28.0	122.8	57.7	<0.01
Stroop word reading	97.3	11.5	87.2	14.8	ns
Stroop color naming	71.9	15.1	65.9	13.6	ns
Stroop color-word naming	36.8	13.5	33.6	12.1	ns
WCST categories completed	5.7	1.1	3.7	1.8	<0.01
WCST total errors	18.8	16.7	40.9	17.1	<0.01
WCST perseverative responses	10.8	10.1	22.8	10.9	0.01
WCST set failure	0.3	0.7	1.4	0.9	<0.01
**Disease characteristics**					
Duration of symptoms (years)	–	–	10.1	2.9	–
UPDRS motor – On Meds	–	–	25.4	13.5	–
UPDRS motor – Off Meds	–	–	34.1	12.4	–
Levodopa equivalent dose	–	–	1179.7	670.9	–
Antidepressant medications (%)	–	–	21	–	–

*BDI-II, Beck Depression Inventory, Second Edition; AS, Apathy Scale; STAI, State-Trait Anxiety Inventory; MMSE, mini-mental state examination; COWA, Controlled Auditory Word Association Test; WAIS-III, Wechsler Adult Intelligence Scale, Third Edition; WCST, Wisconsin Card Sorting Test; UPDRS, Unified Parkinson’s disease rating scale*.

### Event-Related Potential Stimuli and Task

**Figure 1:**
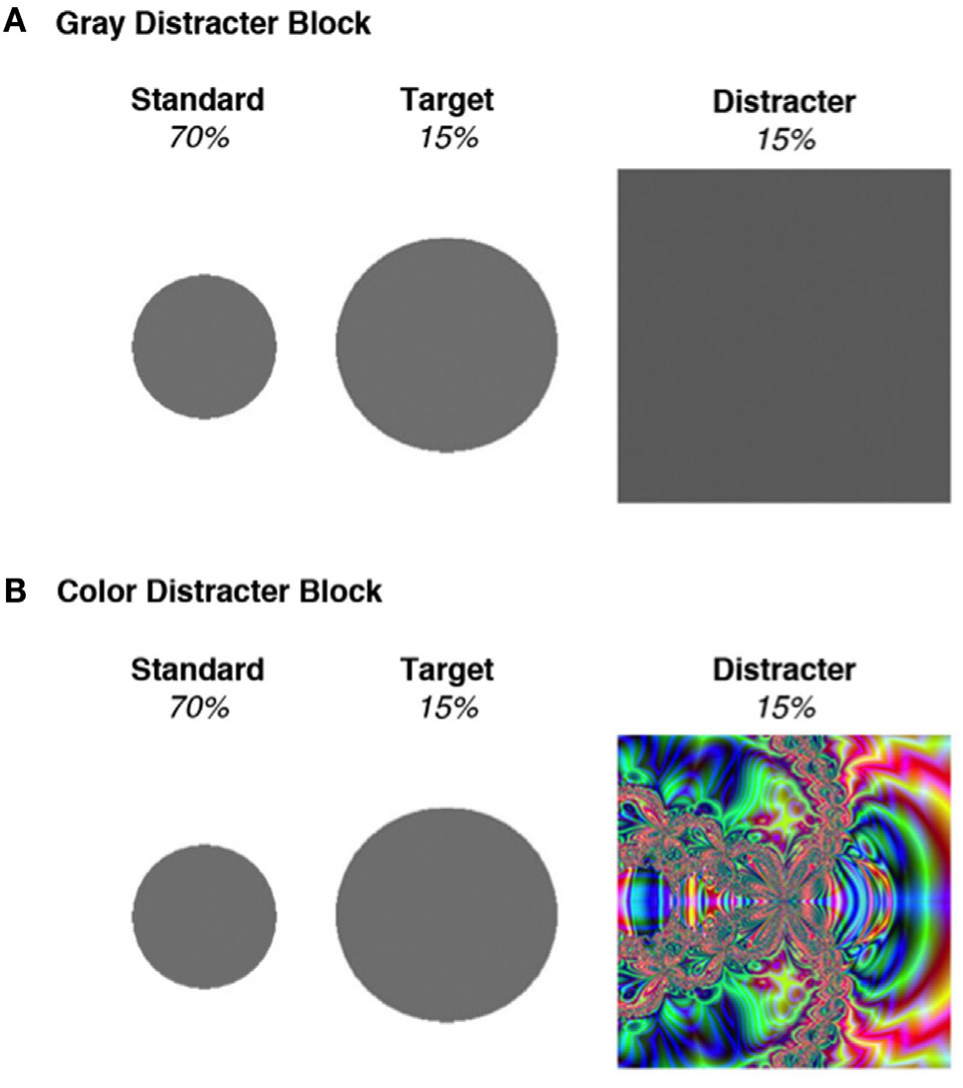
**Stimuli used in the two blocks of the oddball task**. **(A)** All stimuli in the gray distracter block comprised an identical gray color. **(B)** Standards and targets in the color distracter block were identical to those presented in the gray distracter block, but the distracters consisted of unique colorful fractal designs. Adapted from Kaufman et al. (36).

Testing took place in an electrically shielded and sound-attenuated room. All PD patients were evaluated when on their normal dosage of dopaminergic medications. Stimuli and procedures for the three-stimulus oddball task were adapted from Polich and Comerchero (35). Distracter novelty was manipulated by stimulus features contained within the large squares. Half of the distracters were gray, like the targets and standards, and were identical in appearance throughout the experiment. The other distracters contained colorful fractal designs that were unique and only occurred once over the course of the experiment. Mean (±SD) luminance intensity for the color distracter slides was 24.1 ± 9.3 cd/m^2^, which did not deviate substantially from that of gray distracters (23.1 cd/m^2^). Following a brief practice trial, a total of 600 stimuli were randomly presented in four blocks of 150. Each stimulus was presented for 75 ms, with a 2-s inter-stimulus interval. Color and gray distracters were presented in different (counterbalanced) trial blocks to allow the opportunity to examine the block-related effects of distracter type on behavioral and electrophysiological data. See Figure 1 for a sample color distracter and an overview of the stimulus probabilities.

### Electrophysiological Data Recording, Reduction, and Measurement

Electroencephalogram data were recorded from the scalp using a 64-channel system (Electrical Geodesics, Inc., Eugene, OR, USA) running Net Station software. Impedance of electrodes was maintained below 50 kΩ, as recommended by the manufacturer. EEG was initially referenced to the vertex and recorded continuously at 250 Hz, with on-line band-pass filtering from 0.1 to 100 Hz. EEG data were then re-referenced to a right mastoid reference off-line and digitally low-pass filtered at 30 Hz. Following recording, EEG data were adjusted for movement, electromyographic muscle artifact, electro-ocular eye movement, and blink artifacts using spatial filtering methods implemented through Brain Electric Source Analysis [BESA; (37)]. See Figure S1 in Supplementary Material for topography of blink artifacts. All EEG data above 150 μV was discarded, and the maximum allowable amplitude settings for each trial were individualized for each participant [as recommended by (38)]. The average (±SD) amplitude used for rejection was 111 μV (±14), and this threshold did not differ between PD patients and controls [*t*(24) = 1.1, *p* > 0.30]. Point-to-point transitions were not allowed to exceed 75 μV. Of the 64 electrodes used to collect data, a small number of electrodes were interpolated during analysis in order to reduce electrode noise. PD patients required greater electrode interpolation than controls [*t*(24) = 3.2, *p* < 0.01], which is likely due to additional movement artifact caused by Parkinsonian tremors during the EEG recording. Nevertheless, PD patients as a whole required minimal electrode interpolation (8% of electrodes, on average).

Individual-subject stimulus-locked averages were derived separately for standard, target, and distracter stimuli in each of the two distracter blocks. Epochs were extracted from a window of 200 ms prior- to 800 ms post-stimulus presentation and baseline-corrected (200 ms prior to stimulus onset) before subject averaging and analysis. Four midline electrodes corresponding with FCz, Cz, CPz, Pz were chosen for measurement and analysis of P3 responses, as these electrodes demonstrated the maximal amplitude of this component. Four centrofrontal electrodes were chosen to examine anterior P3 difference waves. The time window used for scoring the P3 component was 300–650 ms.

### Data Analysis

Due to a positive skew in the accuracy data, we applied the arcsine transformation for all error rate data (36) and subjected them to a Group (PD, control) × Distracter (gray, color) × Stimulus (standard, target, distracter) repeated measures analysis of variance (ANOVA). For analyses of oddball task response time (RT), median RTs were employed for correct responses (39) and compared between distracter conditions via dependent sample *t*-tests.

Because of their broad scalp distribution, P3 mean amplitudes and peak latencies were examined with Group (PD, control) × Distracter (gray, color) × Stimulus (standard, target, distracter) × Electrode Site (FCz, Cz, CPz, Pz) repeated measures ANOVAs. All other ERP components were subjected to Group (PD, control) × Distracter (gray, color) × Stimulus (standard, target, distracter) repeated measures ANOVAs performed on the electrode location(s) in which each ERP component showed maximal amplitude (40). ANOVAs employed planned contrasts to examine main effects and interactions, with effect sizes reported as partial eta-squared (η^2^). For ANOVAs examining effects of stimulus type, Helmert contrasts first compared standards with targets and distracters, and then compared targets with distracters. For P3 ANOVAs examining effects of midline electrode site, Helmert contrasts compared electrodes in a posterior-frontal progression (Pz, PCz, Cz, FCz).

## Results

### Oddball Task Performance

Behavioral data from the oddball task are presented in Table 2. Planned contrasts revealed a main effect of stimulus type [*F*(1,24) = 9.5, *p* < 0.01, η^2^ = 0.28], such that error responses to distracters (false alarms) and targets (misses) were more common than errors to standards. Distracter type significantly interacted with stimulus type [*F*(1,24) = 12.6, *p* < 0.01, η^2^ = 0.35], as false alarms to distracters were more common than target misses during trial blocks presenting colorful distracters. There was a main effect of group, in which PD patients committed more errors overall than controls [*F*(1,24) = 20.5, *p* < 0.001, η^2^ = 0.46]. Group also interacted with distracter type [*F*(1,24) = 5.8, *p* < 0.05, η^2^ = 0.20], such that controls’ accuracy was greater than PD patients in the gray distracter block. Target RT data did not differ as a function of either group or distracter type (*p*s > 0.5).

**Table 2:** **Behavioral data from the oddball task**.

	Controls		PD patients	
	Mean	SD	Mean	SD
**Gray distracter**				
Reaction time to targets (ms)	473.2	70.9	478.0	103.6
Target response errors (%)	1.8	2.4	10.0	10.6
False alarm to distracters (%)	0.4	0.9	5.4	10.4
False alarm to standards (%)	0.2	0.3	4.7	9.3
**Color distracter**				
Reaction time to targets (ms)	460.6	74.7	491.1	73.5
Target response errors (%)	1.5	2.7	5.3	7.7
False alarm to distracters (%)	4.6	0.6	5.9	3.7
False alarm to standards (%)	0.4	0.5	2.3	4.5

### Event-Related Potential Data

Within the gray distracter block, standard stimuli waveforms contained an average (±SD) of 163.7 ± 28.4 trials, while target waveforms contained 32.9 ± 8.1 trials and distracter waveforms contained 34.8 ± 6.8. Within the color distracter block, standard stimuli waveforms contained an average (±SD) of 173.5 ± 18.8 trials, while target waveforms contained 34.4 ± 7.0 trials and distracter waveforms contained 36.2 ± 4.7. PD patients and controls did not differ in their number of trials that were acceptable for analysis.

#### P3 Amplitude and Latency

**Figure 2:**
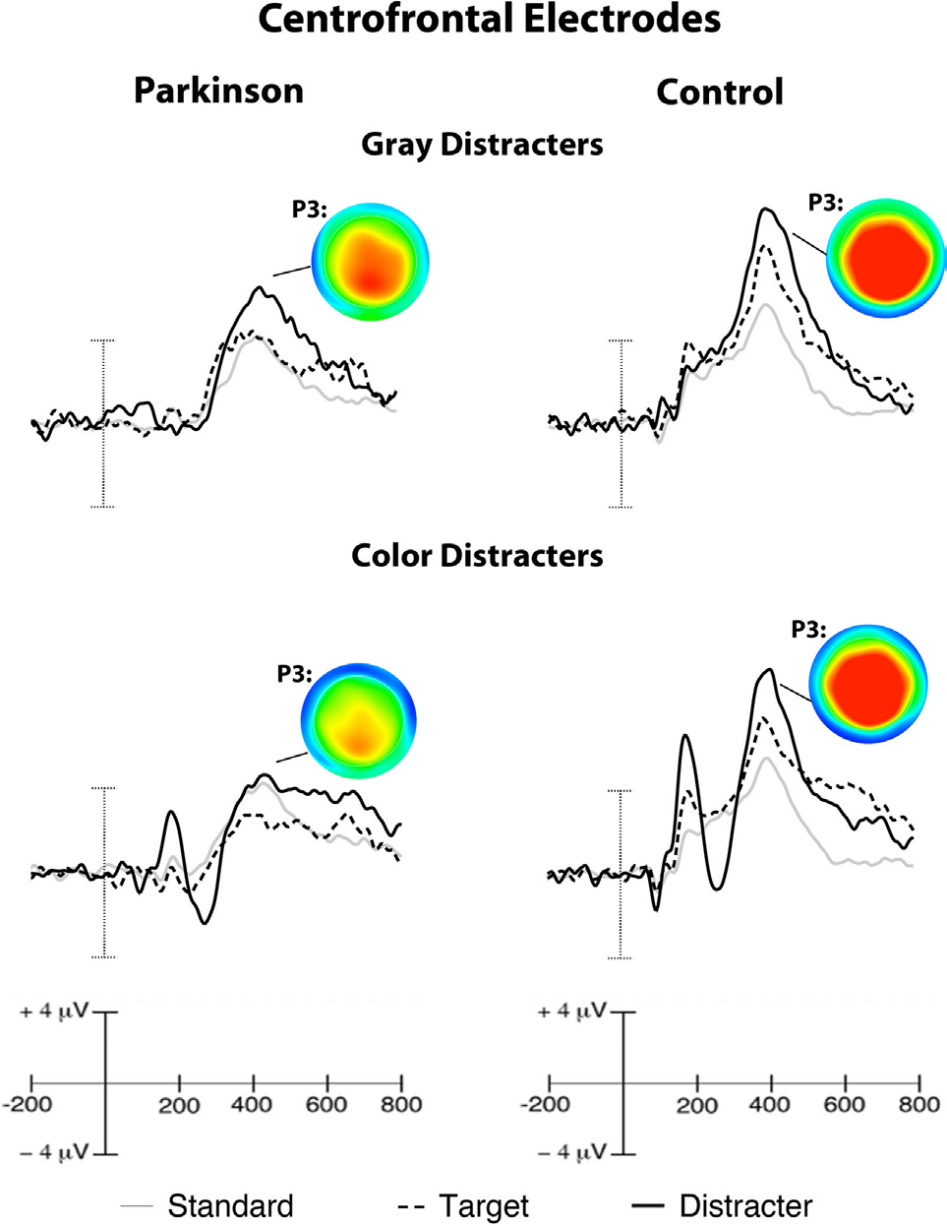
**Grand-averaged ERPs from centrofrontal electrodes**. Scalp maps illustrate peak P3 amplitudes evoked by distracters, which show a centrofrontal reduction in color distracter processing in PD patients. Target P3 amplitudes peaked in parietal electrodes (ERPs not shown). Note: voltage ranges for scalp maps were identical for each group (−5 to +7.5 μV).

P3 activity had a broad distribution for healthy controls, while PD patients showed marked reductions in centrofrontal P3 amplitudes for distracters and targets presented during the color distracter block. Peak P3 potentials for targets were seen in parietal regions. Relative to controls, PD patients exhibited reduced centrofrontal P3 amplitude for distracters relative to targets and standards in the color distracter block [*F*(1,24) = 5.9, *p* < 0.05, η^2^ = 0.20]. This interaction resulted from attenuated distracter-related centrofrontal P3 amplitude for PD patients relative to controls in the color distracter block, as shown in Figure 2.

Midline P3 latencies peaked between 350 and 500 ms across conditions. Over central sites, P3 latencies were shorter for distracters relative to other stimuli [*F*(1,24) = 4.4, *p* < 0.05, η^2^ = 0.15]. Overall P3 latencies were longer for PD patients than controls over central sites [*F*(1,24) = 9.2, *p* < 0.01, η^2^ = 0.28].

#### P3 Difference Waves and Clinical Symptoms in PD

**Figure 3:**
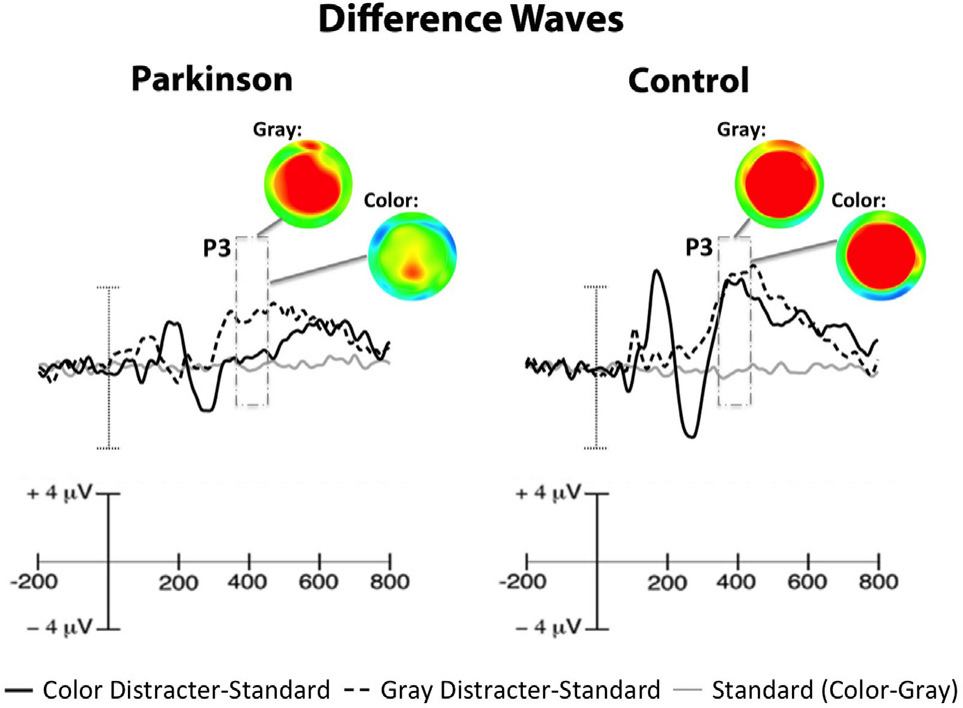
**Centrofrontal difference waves, which show amplitude contrasts for distracter-standard conditions (dark and dotted lines) and standard stimuli taken from color and gray distracter blocks (gray line)**. ERPs to standard stimuli did not differ as a function of distracter color (flat line), so effects seen in distracter-standard difference waves were attributable to the distracters. Scalp maps illustrate distracter-standard P3 difference waves (i.e., P3a), revealing a centrofrontal reduction in color-related distracter processing in PD patients. Note: voltage ranges for scalp maps were identical for each group (−4 to +2 μV).

Even though significant P3 group differences were observed for colorful distracter stimuli, standard stimuli did not demonstrate P3 effects of group (PD mean = 4.24 ± 2.65 μV; Control mean = 4.47 ± 2.61 μV) or distracter block (Color mean = 4.33 ± 2.67 μV; Gray mean = 4.37 ± 2.50 μV). In order to better isolate P3 signals (i.e., P3a) associated with color distracter processing in PD patients, color distracter-standard difference waves were calculated. The resulting difference wave was then scored for mean amplitude during the time interval corresponding to maximal P3 amplitude (380–420 ms). Distracter-standard difference waves are shown in Figure 3.

Color distracter-standard P3 amplitudes (from now on referred to as color P3) were found to correlate with all emotional symptom scales in PD patients, as shown in Table 3. Because of the high degree of intercorrelations between emotional measures, partial correlations were performed such that each emotional measure was correlated with color P3 amplitudes, while controlling for all other emotional measures. Of these partial correlations, only apathy remained significantly correlated with color P3 in PD patients (*r* = −0.65, *p* < 0.05).

**Table 3:** **Intercorrelations for centrofrontal distracter-related P3 amplitude and emotional symptoms in PD patients**.

Measure	1	2	3	4	5
1. Color P3 difference wave	–				
2. Apathy Scale	−0.80***	–			
3. BDI-II	−0.64*	0.90***	–		
4. STAI – trait Anxiety	−0.79*	0.88***	0.91***	–	
5. STAI – state Anxiety	−0.83***	0.77**	0.80***	0.83***	–

*p < 0.05.

**p < 0.01.

***p < 0.001.

**Figure 4:**
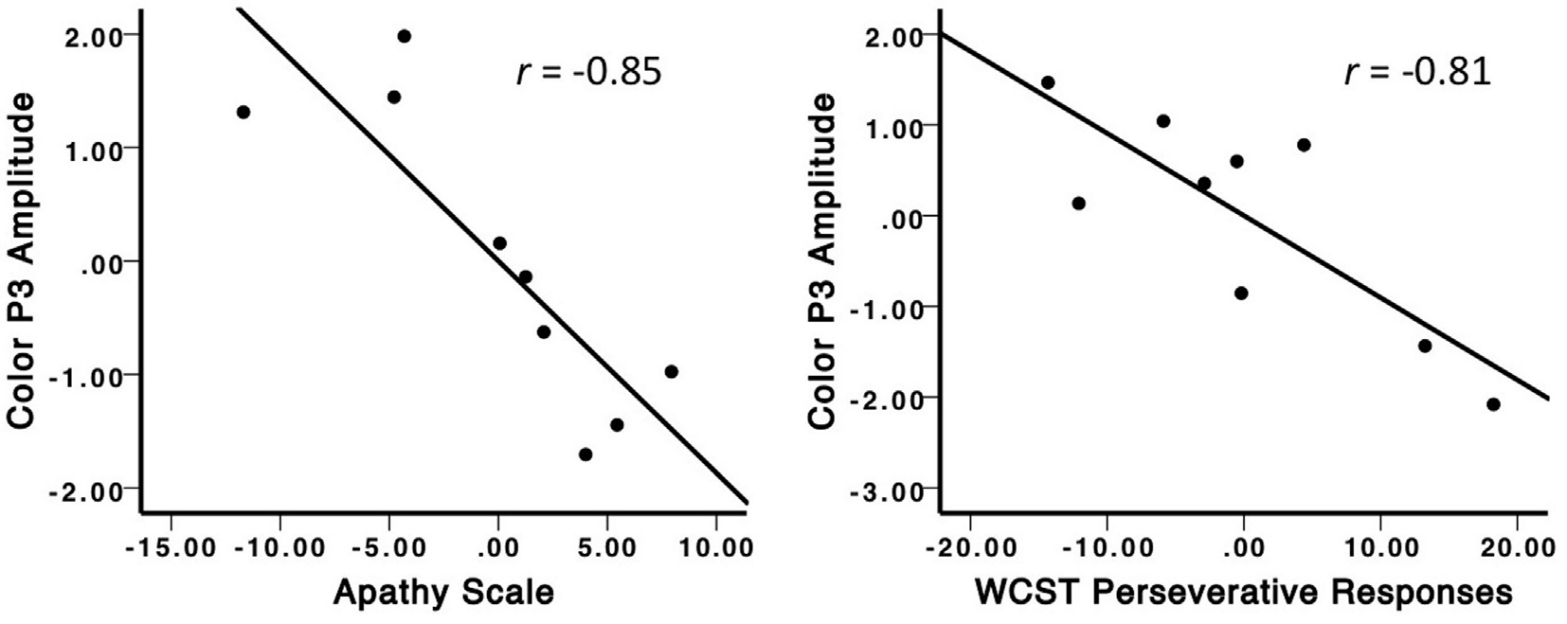
**Partial correlation plots illustrating the unique relationships between color distracter-related P3 amplitude and apathy (left) and WCST perseverative responses (right)**. The negative relationship between color P3 amplitude and apathy was significant, even after controlling for the effects of other emotional scales and executive function. The negative relationship between color P3 amplitude and WCST perseverative responses was significant, even after controlling for the effects of apathy and other executive function measures.

Color P3 amplitudes were also found to correlate with several executive function measures in PD patients, as shown in Table 4. Apathy scores also correlated with these measures of executive function. Partial correlations revealed that WCST perseverative responses remained significantly correlated with color P3 amplitudes, even after controlling for the other executive function measures and apathy scores (*r* = −0.81, *p* = 0.05). Furthermore, partial correlations between apathy scores and color P3 amplitudes also remained significant, after controlling for the executive function measures (*r* = −0.84, *p* < 0.05). These significant partial correlation plots can be seen in Figure 4.

**Table 4:** **Intercorrelations for centrofrontal distracter-related P3 amplitude, executive function, and apathy in PD patients**.

Measure	1	2	3	4	5
1. Color P3 difference wave	–				
2. Trails B	−0.49^t^	–			
3. Stroop color-word	0.69*	−0.62^t^	–		
4. Wisconsin perseverative responses	−0.73**	0.26	−0.67*	–	
5. Apathy Scale	−0.80***	0.69**	−0.73**	0.56*	–

*^t^p < 0.10*.

*p < 0.05.

**p < 0.01.

***p < 0.001.

Parkinson’s disease motor symptoms (e.g., disease duration, symptom severity) and patient age did not significantly correlate with color P3 amplitudes. Similarly, measures of oddball task performance (i.e., target RT, target, and distracter accuracy) did not correlate with color P3 amplitudes. Furthermore, gray distracter-standard P3 amplitudes were not correlated with any of the clinical measures in PD patients. Additionally, none of these variables correlated with color P3 amplitudes in controls.

## Discussion

In this study, we examined ERP reflections of distracter novelty in order to test the hypothesis that heightened levels of apathy would be associated with decreased visual novelty processing in a heterogeneous sample of PD patients. In support of our hypothesis, this study revealed three major findings. First, PD patients exhibited reduced distracter-related ERPs over centrofrontal electrode sites, indicating disruptions in attentional orienting toward novelty. Second, apathy symptoms were strong predictors of these P3 potentials, such that elevations in apathy corresponded with reductions in P3 amplitude across all PD patients. Although other emotional symptoms also correlated with P3 amplitude, these relationships disappeared when controlling for apathy. Third, executive functioning in PD independently correlated with distracter-related P3 potentials; however, apathy remained a significant predictor even when accounting for executive function.

### Novelty and P3 Potentials

Our finding of reduced centrofrontal P3 amplitudes in PD patients is consistent with earlier research (25), supporting the notion that PD patients exhibit dysfunction in novelty processing that can be observed as a centrofrontal electrophysiological deficit. The fact that these effects were sensitive to distracter content (i.e., lowest P3 amplitudes for unique color patterns) suggests that PD patients have disproportionate difficulty processing visual information that is both distracting and highly novel. Although it was predicted that distracter-related P3 activity would correlate with apathy symptoms in PD patients, it was not expected that emotional symptoms as a whole would be so highly associated with color P3 potentials. While these findings may suggest that the novelty processing in PD is associated with a broadly defined state of negative affect, the core emotional symptom driving this effect was shown to be apathy.

Similar to other previous findings (19), several executive functioning measures were also correlated with reductions in color P3 amplitudes in PD. This allowed us to examine the degree to which apathy and executive functioning could be distinguished in their contributions to P3 amplitude. When controlling for the overall variance among these measures, apathy and perseverative errors on the WCST demonstrated unique relationships with color P3 amplitude, revealing that both emotional and executive symptoms are closely related to novelty processing deficits in PD. Although previous studies have found relationships between WCST performance and distracter-related P3 amplitudes in PD (19, 25), our findings are the first to show that apathy and executive function are independently linked to novelty processing in this population.

### Implications of Novelty Processing Deficits in PD

The strong association between apathy symptoms and novelty processing suggests that the neural systems underlying motivation and attention are intricately linked in PD. This connection is consistent with our current understanding of apathy. Marin (16) regarded apathy as a primary deficit in motivation and goal-directed behavior. Although novelty processing is often conceptualized as an automatic reflex that orients attention toward unexpected events, novelty processing can be vulnerable to top-down modulation.

Using a three-stimulus oddball task similar to the one involved in this project, Chong et al. (41) included a condition in which participants were instructed to visually explore the novel stimuli as task-relevant “invitations” for further processing, rather than ignoring them as task-irrelevant distracters. This condition led to enhanced novelty processing, as reflected in larger P3 amplitudes, and provided evidence that changes in context – both experimentally and personally derived – can have dramatic changes on how novel information is processed. One can speculate that apathy symptoms in the PD patients had the opposite effect of Chong et al.’s invitation to visually explore the novel distracters. In the current study, PD patients did not selectively ignore these stimuli, as false alarm rates were not significantly different between color and gray distracters. However, it appears that their attentional orienting mechanisms failed to fully “explore” these novel events in a manner that is consistent with their self-reported symptoms of apathy.

### Limitations

Several limitations of the present study need to be acknowledged. The PD sample was variable with regard to disease duration, yet most of the PD patients were in H-Y stages 2–3. Patients were tested on dopaminergic medications, so specific effects of medication could not be addressed in the current study. While PD patients were more depressed and anxious than controls, they did not endorse greater apathy symptoms as a whole. As a result, only a small number of PD patients in this study were suffering from clinical levels of apathy. Nevertheless, the strong correlations between apathy and P3 amplitudes seen across all patients demonstrate the utility of examining apathy in a continuous manner. While apathy cut-off scores are meaningful for characterizing the clinical level of apathy severity in PD patients, our results show that levels of apathy need not be excessive in order to show strong associations with novelty processing. Other limitations are that the PD sample was well educated, predominantly male, and variable in age (43–77 years). As a result, these findings may not generalize to all PD patients. However, the variability observed in age, disease duration, and symptom severity did confound the results of this study, as these variables were not associated with emotional symptoms, cognitive symptoms, or distracter-related P3 potentials.

## Conclusion

In summary, our findings support the hypothesis that apathy and executive functioning in PD are both strongly associated with deficits in novelty processing. PD patients showed reduced processing of novel distracters, as measured by the amplitude of centrofrontal distracter-related P3 components. Distracter-related P3 amplitudes were strongly associated with apathy symptoms, even after controlling for the effects of depression, anxiety, and executive function. These findings help to clarify the psychophysiological implications of apathy, and may help facilitate a more biological understanding of this common symptom of PD. The combined effects of apathy, executive dysfunction, and novelty processing have far-reaching implications for goal-directed behavior in this population. Failure to orient attention appropriately may leave PD patients lost in a world of constantly changing attentional demands. These core deficits in attention may strip PD patients of the cognitive and motivational tools needed to flexibly respond to their environments. Further research is needed to better characterize the neural basis of these findings and determine how these deficits can be optimally managed to improve quality of life in this clinical population.

## Ethics Statement

Study approved by the University of Florida, Health Sciences Institutional Review Board. Participants were provided with an informed consent form, which allowed them to understand the nature of the study, along with risks and benefits of participating. After reading the consent form and having an opportunity to ask questions of the research team, participants provided written consent to participate.

## Author Contributions

DK completed data collection, analysis, and manuscript preparation. DB assisted with experimental design, patient recruitment, and manuscript preparation. MO assisted with patient recruitment and manuscript preparation. RP assisted with manuscript preparation. WP assisted with experimental design, data analysis, and manuscript preparation.

## Conflict of Interest Statement

The authors declare that the research was conducted in the absence of any commercial or financial relationships that could be construed as a potential conflict of interest.
