# Supplementary material for: Apathy, Novelty Processing, and the P3 Potential in Parkinson’s Disease
Source: Front Neurol. 2016 Jun 23;7:95. doi: 10.3389/fneur.2016.00095 (PMC4917554; doi:10.3389/fneur.2016.00095)
Supplement: Supplementary file 1 [file Image_1.pdf]

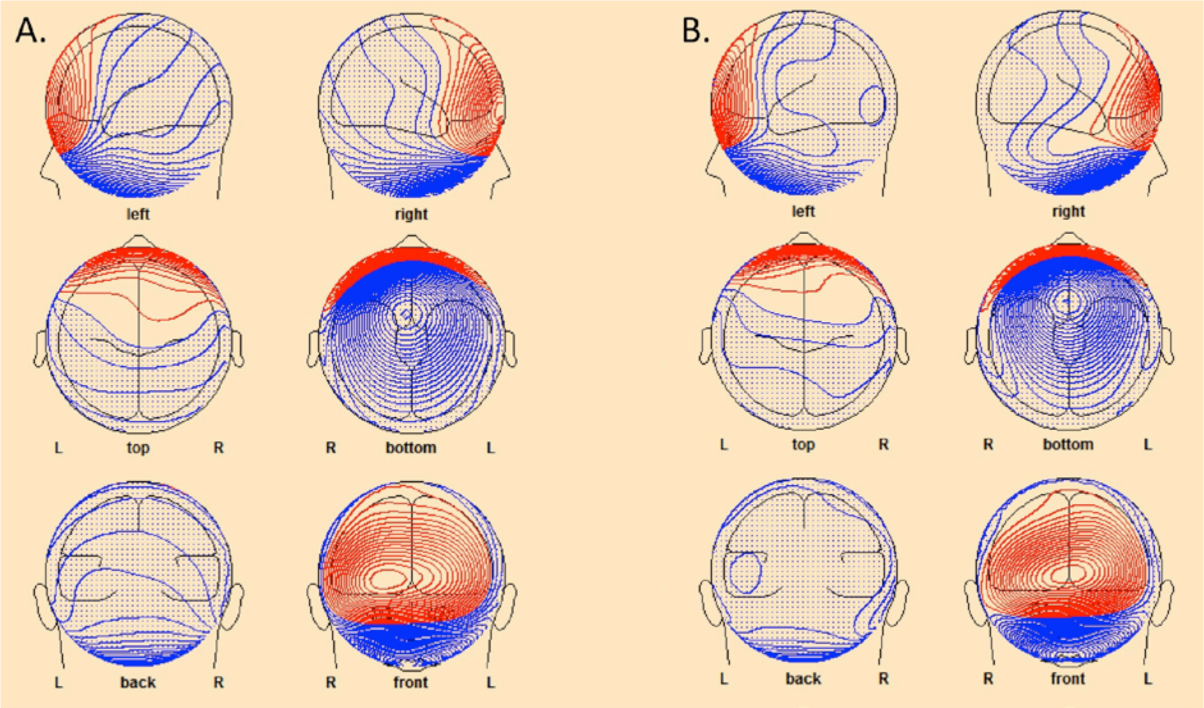

FIGURE S1 | Scalp maps illustrating the PCA topography of blink artifacts, as revealed by spatial filtering methods. (A) Sample control participant's blink artifact. **(B)** Sample PD patient's blink artifact. Note: blink patterns were associated with a large frontal positivity (red) that was picked up by frontal face and scalp electrodes.
